# Bi‐objective optimization of catheter positions for high‐dose‐rate prostate brachytherapy

**DOI:** 10.1002/mp.14505

**Published:** 2020-10-21

**Authors:** Marjolein C. van der Meer, Peter A.N. Bosman, Yury Niatsetski, Tanja Alderliesten, Niek van Wieringen, Bradley R. Pieters, Arjan Bel

**Affiliations:** ^1^ Department of Radiation Oncology Amsterdam UMC University of Amsterdam Amsterdam 1100DD The Netherlands; ^2^ Life Sciences and Health research group Centrum Wiskunde & Informatica Amsterdam 1098XG The Netherlands; ^3^ Physics and Advanced Development Elekta Veenendaal 3900AX The Netherlands; ^4^ Department of Radiation Oncology Leiden University Medical Center Leiden 2300RC The Netherlands

**Keywords:** bi‐objective optimization, catheter positions, HDR brachytherapy, prostate neoplasms, treatment planning

## Abstract

**Purpose:**

Bi‐objective simultaneous optimization of catheter positions and dwell times for high‐dose‐rate (HDR) prostate brachytherapy, based directly on dose‐volume indices, has shown promising results. However, optimization with the state‐of‐the‐art evolutionary algorithm MO‐RV‐GOMEA so far required several hours of runtime, and resulting catheter positions were not always clinically feasible. The aim of this study is to extend the optimization model and apply GPU parallelization to achieve clinically acceptable computation times. The resulting optimization procedure is compared with a previously introduced method based solely on geometric criteria, the adapted Centroidal Voronoi Tessellations (CVT) algorithm.

**Methods:**

Bi‐objective simultaneous optimization was performed with a GPU‐parallelized version of MO‐RV‐GOMEA. This optimization of catheter positions and dwell times was retrospectively applied to the data of 26 patients previously treated with HDR prostate brachytherapy for 8–16 catheters (steps of 2). Optimization of catheter positions using CVT was performed in seconds, after which optimization of only the dwell times using MO‐RV‐GOMEA was performed in 1 min.

**Results:**

Simultaneous optimization of catheter positions and dwell times using MO‐RV‐GOMEA was performed in 5 min. For 16 down to 8 catheters (steps of 2), MO‐RV‐GOMEA found plans satisfying the planning‐aims for 20, 20, 18, 14, and 11 out of the 26 patients, respectively. CVT achieved this for 19, 17, 13, 9, and 2 patients, respectively. The *P*‐value for the difference between MO‐RV‐GOMEA and CVT was 0.023 for 16 catheters, 0.005 for 14 catheters, and <0.001 for 12, 10, and 8 catheters.

**Conclusions:**

With bi‐objective simultaneous optimization on a GPU, high‐quality catheter positions can now be obtained within 5 min, which is clinically acceptable, but slower than CVT. For 16 catheters, the difference between MO‐RV‐GOMEA and CVT is clinically irrelevant. For 14 catheters and less, MO‐RV‐GOMEA outperforms CVT in finding plans satisfying all planning‐aims.

## 1 INTRODUCTION

Treatment planning for high‐dose‐rate (HDR) prostate brachytherapy consists of two parts. First, the positions of the catheters to be placed inside the patient have to be determined. For this, either (semi‐)manual planning or inverse planning can be used at the time of catheter placement. A commonly used (semi‐)manual planning approach is the peripheral loading technique where the majority of the catheters is placed at the periphery of the prostate with relatively few catheters close to the urethra, used in our hospital. Typically 14–20 catheters are used, depending on the prostate size. Alternatively, a commonly used inverse planning approach is hybrid inverse planning optimization (HIPO).^1^

Second, once the catheters are placed, the dwell times for the source dwell positions have to be determined. For this, current practice is to optimize the dwell times using automated methods such as the inverse planning simulated annealing (IPSA)^2^ or more recently the hybrid inverse planning optimization (HIPO)^1^ algorithms. Other methods have been investigated for optimizing the dwell times directly on dose‐volume indices,^3^, ^4^, ^5^ as well as (dose‐volume based) approaches that consider multiple objectives.^6^, ^7^, ^8^, ^9^, ^10^ A multi‐objective optimization method that directly optimizes dose‐volume indices has been proposed by leveraging the Multi‐Objective Real‐Valued Gene‐pool Optimal Mixing Evolutionary Algorithm (MO‐RV‐GOMEA).^11^ This method was shown to be capable of outperforming both the current clinical practice of dwell time optimization^12^ as well as parameter tuning for IPSA and HIPO.^13^

The model optimized with GOMEA is bi‐objective and gives insight into the trade‐off between covering the targets and sparing the organs at risk (OARs). However, the quality of the treatment plans that can be achieved in dwell time optimization only, depends on the catheter positions. Optimizing not only the dwell times, but also the catheter positions could therefore have added value over optimizing only dwell times. In literature, two different approaches for catheter position optimization have been described.^14^

The first approach is to optimize the catheter positions independent of the dwell times,^15^, ^16^, ^17^ based solely on geometrical properties of target and organs at risk shapes, without clinical evaluation criteria such as dose‐volume indices. Such approaches have been shown to perform well when compared to the clinical treatment plans. Among such approaches, especially the adapted Centroidal Voronoi Tessellations (CVT) algorithm has shown promising results,^17^ outperforming existing optimization methods^18^ such as HIPO catheter position optimization. In CVT, 3D organ shapes are projected onto a plane as 2D contours, and catheter positions are optimized solely based on their distribution in these projections. The disadvantage of this approach is that the objectives of catheter position optimization and the objectives of dwell time optimization are decoupled. For catheter position optimization, the distribution of the catheter positions in the organ projections is evaluated, whereas for dwell time optimization, properties of the dose distribution are evaluated, which are ultimately the most important. Therefore, the overall optimal catheter positions may not be found.

The second approach is to simultaneously optimize catheter positions and dwell times.^1^, ^19^, ^20^, ^21^ The advantage of this approach is that the objectives are identical to the case of optimizing only dwell times. The disadvantage is that a simultaneous approach often requires large computation efforts, especially when optimizing directly on dose‐volume indices. In particular, straightforwardly extending GOMEA to also optimize catheter positions requires several hours of runtime.^21^ Since determining catheter positions is part of the preplanning done in the operating room, these runtimes are not clinically feasible. Moreover, preliminary results of previous research showed that use of the optimization model (also used for only dwell times) did not guarantee finding catheter positions that would be acceptable to radiation oncologists: in particular, hot spots in the healthy tissue were observed, and catheters were placed too close to OARs.^21^

In this study, we improve on GOMEA prostate catheter position optimization^21^ to obtain a clinically feasible optimization method. First, we extend the previously used optimization model to ensure treatment plans are found that are acceptable to radiation oncologists. Second, we apply parallelization on a Graphics Processing Unit (GPU) to speed up the computation and achieve clinically acceptable runtimes. We compare the results of GOMEA catheter position optimization with CVT,^17^ to study the possible advantages and disadvantages of simultaneous optimization of both catheter positions and dwell times, with respect to the geometry‐based method CVT.

## 2 BACKGROUND

### 2.A Bi‐objective optimization model

The bi‐objective optimization model is based directly on the dose‐volume indices in the clinical protocol. In general, there is an intuitive key trade‐off in a given clinical protocol between the planning‐aims for the targets (i.e., prostate and seminal vesicles should receive enough dose) and those for OARs (i.e., bladder, rectum, and urethra should not receive too much dose). This can be captured by using two objectives, i.e., a bi‐objective approach. Optimization then does not result in a single treatment plan, but in a set of plans, each of which has a different trade‐off between the two objectives. This set of optimized plans is called the approximation set and their corresponding objective values make up the approximation front, as it is an approximation of the Pareto front that contains all theoretically (Pareto‐) optimal solutions. The physician can choose a plan from this front, potentially using additional patient information such as age and previous treatments.

The bi‐objective optimization model is based on the clinical protocol for HDR prostate brachytherapy at the Amsterdam UMC. Before undergoing brachytherapy, patients have received 20 External Beam Radiation Therapy (EBRT) fractions of 2.20 Gy. The planning‐aim dose for brachytherapy is 13 Gy (terminology based on the ICRU 89 report,^22^ often called prescribed dose). The constraints on the EQD2 of the combined treatment are $D_{1\text{cm}3}^{\text{bladder}}<\;78\;\text{Gy}$, $D_{2\text{cm}3}^{\text{bladder}}<\;70\;\text{Gy}$, $D_{1\text{cm}3}^{\text{rectum}}<\;73\;\text{Gy}$, $D_{2\text{cm}3}^{\text{rectum}}<\;70\;\text{Gy}$, and $D_{0.1\text{cm}3}^{\text{urethra}}<\;95\;\text{Gy}$. Using an α/β‐ratio of 3, the resulting constraints on the brachytherapy are shown in Table I. The dose‐volume indices of the protocol are combined into two objectives to be maximized, the Least Coverage Index (LCI) and the Least Sparing Index (LSI).

**Table I:** The high‐dose‐rate (HDR) prostate brachytherapy planning protocol at the Amsterdam UMC.

Targets		OARs		
Prostate	Seminal vesicles	Bladder	Rectum	Urethra
$V_{100\%}\;>\;95\%$	$V_{80\%}\;>\;95\%$	$D_{1\text{cm}3}\;<\;86\%$	$D_{1\text{cm}3}\;<\;78\%$	$D_{0.1\text{cm}3}\;<\;110\%$
$V_{150\%}\;<\;50\%$		$D_{2\text{cm}3}\;<\;74\%$	$D_{2\text{cm}3}\;<\;74\%$	
$V_{200\%}\;<\;20\%$				

Volume indices have an index in percentage of the planning‐aim dose of 13 Gy and a unit in percentage of total organ volume. Dose indices have an index in absolute volume in cm^3^ and a unit in percentage of planning‐aim dose. The seminal vesicles are considered to harbor microscopic disease (in contrast to the macroscopic disease in the prostate), hence 80% of the planning‐aim dose is sufficient.

### 2.B Organ reconstruction settings

The 3D organ shapes are reconstructed from delineated 2D organ contours. The organ reconstruction settings are based on our clinically used treatment planning system, Oncentra Brachy v4.5. In particular:

1. The urethra is considered as part of the organs it intersects;
2. Contour interpolation is used to add interpolated contours between each pair of delineated contours (which was performed on each slice);
3. Most caudal/cranial contours span the half‐slice thickness in the direction of the other contours of that organ.

The resolution of the slices (craniocaudal direction) is lower than the in‐plane resolution and organ surfaces are smoothed in that direction by applying contour interpolation.^23^ In particular, shape‐based interpolation using a chamfer distance^24^ is used. Since interpolation can be performed in parallel before calculating the dose‐rate matrix, the influence on optimization time is negligible. The impact of these organ reconstruction settings on dose‐volume indices has already been explored in our previous work.^23^

### 2.C Catheter position optimization

**Fig. 1:**
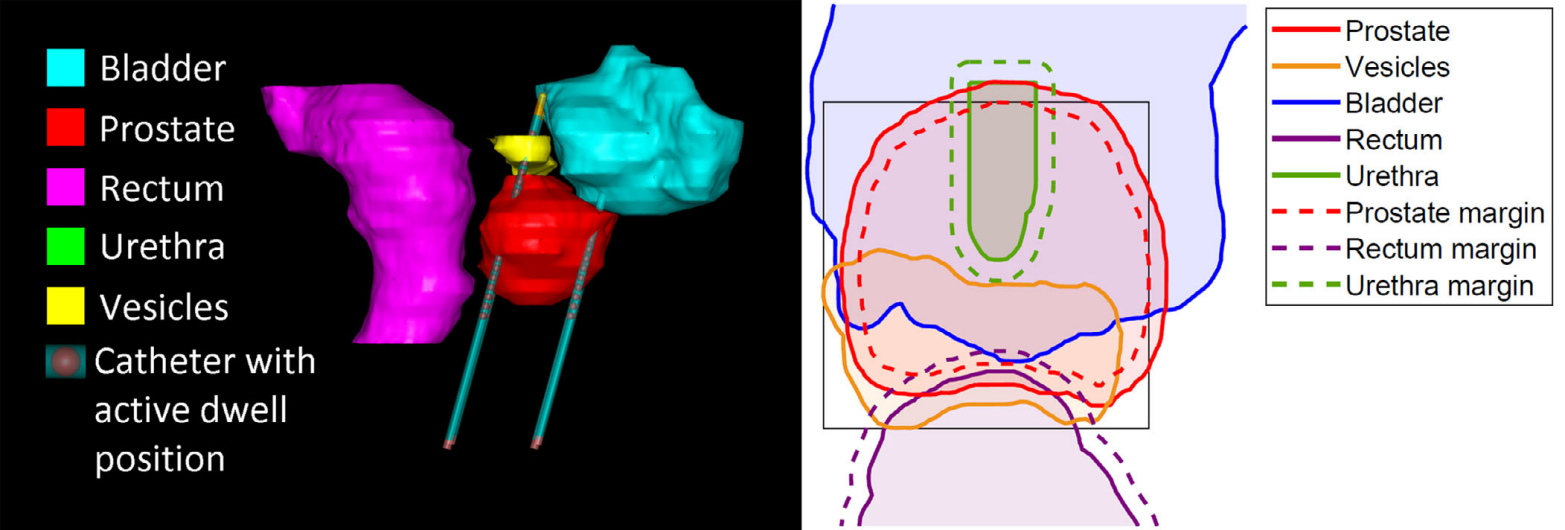
Illustration of the organ projections on a plane parallel to the planes in which the organ contours were delineated (for patient 18). The box defining the catheter grid for GOMEA catheter position optimization is shown in gray lines. Solid lines show the organ projections, dashed lines show the used margins from the organ projections to the centers of the catheter positions (hence taking into account the radius of a catheter). [Color figure can be viewed at wileyonlinelibrary.com]

The orientations of the catheters are modeled to be parallel in relation to each other, following the usual clinical implantation procedure. The feasibility of catheter positions is checked by projecting all organs and catheters onto a plane parallel to the planes in which the organ contours were delineated (Fig. 1), along the direction of the catheters.

The positions of the catheters during optimization are unrestricted, in the sense that no fixed template of catheter positions is used. However, at the start of optimization, a fine two‐dimensional rectangular grid of parallel catheters is placed over the organ projection plane. During optimization, nearest neighbor interpolation is used on the catheter positions with respect to the catheter positions in the grid, effectively limiting catheter positions to these grid positions. The advantage of this grid is that dose‐rates only have to be calculated at the beginning of optimization, speeding up the overall optimization process. By using a very fine grid, all catheter positions can effectively still be chosen. This is in accordance with clinical practice at the Amsterdam UMC, where a catheter can be placed at any desired position by using a template enabling movement of the catheter‐guidance arm in lateral and ventro‐dorsal direction.

The size of the grid is set depending on the shape of the targets. A bounding rectangle around the projections of prostate and seminal vesicles is determined, with length dX and width dY. Then, for a grid of up to G catheters, we define the grid as $\left\lfloor \sqrt{G\cdot dX/dY}\right\rfloor$ by $\left\lfloor \sqrt{G\cdot dY/dX}\right\rfloor$ catheters. Once the grid is placed, dose‐calculation points are sampled in the targets, OARs, and healthy tissue. Next, a dose‐rate matrix is constructed, containing the dose‐rate from each dwell position in each of the grid catheters to each of the sampled dose‐calculation points.

### 2.D MO‐RV‐GOMEA

MO‐RV‐GOMEA is an evolutionary algorithm. As such, it maintains a set of potential solutions during optimization, rather than a single solution. This set is called the population and the number of potential solutions is called the population size.

An important feature of GOMEA is its ability to exploit dependencies between variables. This is typically done through a so‐called linkage tree.^25^ The bottom of this tree consists of all sets containing only a single variable. Hence, for n variables $x_{1},\ldots ,x_{n}$, n sets $\left\{x_{i}\right\}$ for i=1,…,n are created. Next, sets with variables that are highly dependent on each other are repeatedly merged, until the top set with all variables $\left\{x_{1},\ldots ,x_{n}\right\}$ is created, resulting in a total of 2n‐1 sets. The set containing all these sets is called the Family of Subsets (FOS). Therefore, each set on its own is called a FOS element. Dependencies are now exploited by looping over the FOS elements in a random order, iteratively changing variables in a FOS element together, and checking if the change leads to an improvement. This increases the efficiency if strong dependencies are present. Actual changes to variables are made via the estimation and sampling of Gaussian distributions, rendering the algorithm largely derivative‐free. For more details, we refer the interested reader to the literature.^25^

### 2.E CVT

The catheter positions used in clinical practice at the Amsterdam UMC are determined by the radiation oncologists based on experience, in combination with a HIPO generated plan, which is the clinical standard for inverse optimization. However, the adapted CVT algorithm has been shown to outperform HIPO catheter position optimization.^18^ We therefore compare GOMEA with the adapted CVT algorithm.

The adapted CVT algorithm^17^ samples many points inside organ projections and runs a procedure much like the well‐known k‐means clustering procedure to place k catheters. Catheter positions are restricted to be inside the prostate projection, and outside of the urethra and rectum projections (without using margins). The algorithm has three important parameters: the number of catheters, the number of iterations, and the number of points sampled in the organ projections. Following literature,^17^ the number of iterations is set to 100, and the number of sample points is set to 2500. After the catheter positions have been determined, the dwell times can be optimized independently. In previous work, the inverse planning simulated annealing algorithm (IPSA) was used^17^; here, we use GOMEA dwell time optimization^5^ for a fair comparison.

## 3 MATERIALS AND METHODS

### 3.A Optimization model

For the optimization model, the following definitions are used:

**Table:** 

$V_{a\text{\%}\;}^{o}$	Volume of organ o that receives at least a% of the planning‐aim dose of 13 Gy, as percentage of total organ volume.
${\bar{V}}_{a\%}^{o}$	Volume of organ o that receives at least a% of the planning‐aim dose of 13 Gy, in cm^3^.
$D_{a\text{cm}3}^{o}$	Lowest dose to the most irradiated a cm^3^ of organ o, as percentage of the planning‐aim dose of 13 Gy.
N	Number of catheters.
∅	Catheter diameter of 2.35 mm.
$\left(x_{i},y_{i}\right)$	Center of the projection of catheter i.
$t_{k}$	Dwell time k.
$P_{a}^{o}$	Projection of organ o with a mm margin.

The optimization model is as follows:

(1) $$\begin{array}{c} \text{Maximize}\\ \text{LCI}=\min\left\{V_{100\text{\textbackslash{}\%}\;}^{\text{prostate}}‐95,V_{80\text{\textbackslash{}\%}\;}^{\text{vesicles}}‐95\right\} \end{array}$$

(2) $$\text{LSI}=\min\left\{\begin{array}{c} 86‐D_{1\text{cm}3}^{\text{bladder}},74‐D_{2\text{cm}3}^{\text{bladder}},78‐D_{1\text{cm}3}^{\text{rectum}},74‐D_{2\text{cm}3}^{\text{rectum}},110‐D_{0.1\text{cm}3}^{\text{urethra}},\\ 50‐V_{150\text{\textbackslash{}\%}\;}^{\text{prostate}},20‐V_{200\text{\textbackslash{}\%}\;}^{\text{prostate}} \end{array}\right\}$$

(3) $$\begin{array}{c} \text{subject to}\\ C=\left\{\begin{array}{cc} {\bar{V}}_{200\text{\textbackslash{}\%}\;}^{\text{healthy tissue}}‐0.125N, & \text{for LSI}\;\geq ‐25\\ {\bar{V}}_{200\text{\textbackslash{}\%}\;}^{\text{healthy tissue}}‐0.125N\left(1+\frac{‐25‐\text{LSI}}{100}\right), & \text{for LSI}<‐25 \end{array}\right\}\leq 0 \end{array}$$

(4) $$\left(x_{i},y_{i}\right)\in \left(P_{‐\left(\emptyset /2+1\right)}^{\text{prostate}}\;\cup \;P_{0}^{\text{vesicles}}\right)\cap {\left(P_{\emptyset /2+1}^{\text{urethra}}\;\cup \;P_{\emptyset /2+1}^{\text{rectum}}\right)}^{c}$$

(5) $$\begin{array}{c} \sqrt{{\left(x_{i}‐x_{j}\right)}^{2}+{\left(y_{i}‐y_{j}\right)}^{2}}\geq \;\emptyset \;+\;1\forall i\;\neq \;j\\ t_{k}\;\geq \;0\forall k \end{array}$$

Equations (1) and (2) define the Least Coverage Index (LCI) and the Least Sparing Index (LSI).

We have previously shown that when optimizing catheter positions based on the clinical protocol, a constraint is necessary to restrict dose to healthy tissue outside of delineated targets and OARs,^21^ especially since dwell positions outside of the prostate can be used for dose planning. In clinical practice at the Amsterdam UMC, this is usually described in terms of the in‐slice diameter of hot spots in healthy tissue, but this is time‐consuming to calculate during optimization. In contrast, dose‐volume indices such as the V_200%_ can be calculated more quickly and have been applied previously to limit dose to healthy tissue,^26^ but cannot distinguish between multiple small hot spots occurring in a plan with many catheters, and few large hot spots typical for a plan with fewer catheters. We therefore define a constraint on the V_200%_ of the healthy tissue, which is dependent on the number of catheters (N). Our investigations (described in the appendix) have led to the following constraint: ${\bar{V}}_{200\%}^{\text{healthy tissue}}\leq 0.125N$.

If this constraint is added as is to the optimization model, the feasible search space is shrunk to the extent where all initial solutions in the optimization process are infeasible, making optimization unnecessarily more difficult. However, this constraint is mainly important when plans are of sufficient high quality. Hence, the constraint can be relaxed if solutions are still far from optimal, thereby ensuring that initial solutions are also feasible in terms of the optimization model, making optimization itself more efficient and effective. Particularly, we relax this healthy tissue constraint when the LSI is already below a certain threshold. The healthy tissue constraint is used as a hard constraint in the optimization, separate of the LCI and the LSI, to ensure that all treatment plans in the final front satisfy it (Eq. (3)).

Catheters are constrained to be inside the prostate or seminal vesicles, as well as at least 1 mm away from rectum and urethra (measured from the catheter surface). For fixation of catheters in the patient, it is preferable that catheters intersect the prostate, and not just touch the surface. Therefore, catheters have to be at least 1 mm (measured from the catheter surface) away from the edge of the projection of the prostate (Eq. (4)). The minimum distance between catheters (measured from the catheter surface) is 1 mm (Eq. (5)), where the angle between the catheters and the projection plane should be taken into account.

The algorithm contains a parameter describing how far catheters can intersect the bladder. In our experiments, catheters cannot intersect the bladder (including the 5 mm long catheter tip which does not contain dwell positions) but may touch the bladder. A grid size G of 400 catheters is used.

### 3.B GPU parallelization

The GPU parallelization of the simultaneous optimization of catheter positions and dwell times using GOMEA expands previous work focused only on dwell time optimization.^5^ In this approach, sets of treatment plans are evaluated in parallel, and the dose in each dose‐calculation point of each of these treatment plans is calculated on a separate thread. The programming for the GPU was done in CUDA (NVIDIA Corporation, Toolkit v8.0.61) and was run on an NVIDIA Titan Xp GPU, which contains 12 GB of memory. In order to improve efficiency, two algorithmic changes are made as well, which are explained in this section.

In general, the performance of an evolutionary algorithm is dependent on the population size. For dwell time optimization on a GPU, a fixed population size of 96 (a multiple of the selected GPU block size of 16) was reported to lead to good results.^5^ However, catheter position optimization requires a larger population size than dwell time optimization as the search space is both larger and more intricate with strong dependencies between catheter position variables and dwell times. A fixed population size of 300 was reported to lead to good results.^21^ Although automatic population‐sizing schemes are available,^11^ they tend to induce an overhead for the total runtime. As clinical constraints require short computation times, we opted for tuning the population size experimentally to find a good value near 300 that is compatible with the use of a GPU. For catheter position optimization on a GPU, this resulted in the use of a fixed population size of 288 (also with a GPU block size of 16).

Secondly, for dwell time optimization, the dependency between two dwell times is based on the distance between the corresponding dwell positions. After changing the variables in a FOS element, the dose‐volume indices of the treatment plan have to be re‐evaluated. Therefore, FOS elements with only a few variables are relatively expensive: the treatment plan is only slightly changed, but all dose‐volume indices have to be recalculated. Hence, to achieve the shortest runtime of the GPU parallelization, all subsets with <5 variables are removed from the FOS, following previous work.^5^

For catheter position optimization, we compute the distance between catheters and between dwell positions by first computing the average catheter positions over the population. For the two different types of variables, namely catheter position variables and dwell time variables, these distances are used to build two separate Linkage Trees. For the Linkage Tree of the dwell time variables, all subsets with <5 variables are removed. For the Linkage Tree of the catheter position variables, no subsets are removed. The remaining sets of both Linkage Trees are combined into one FOS, to be used for optimization.

### 3.C Patient data

The patient data consisted of 26 patients who consecutively underwent prostate HDR brachytherapy between February 2015 and April 2017. After catheter implantation, MRI scans were acquired with a resolution in the axial planes of 0.52 × 0.52 mm and a slice thickness of 3.3 mm (including a 0.3 mm gap). Reconstruction of catheters and organ contours was performed using manual delineations on most of the axial slices, using the delineations suggested by the interpolation algorithm of the clinical treatment planning system on the rest. Only the base of the seminal vesicles was delineated. Contour interpolation was used to add three interpolated contours between each pair of delineated contours, in line with previous work.^5^ The data on the implanted catheters were not used when performing catheter position optimization.

### 3.D Experiments

The two approaches, GOMEA simultaneous catheter position and dwell time optimization, and CVT catheter position optimization followed by GOMEA dwell time optimization, are compared as follows. Dose‐rate matrices were calculated with the mHDR‐v2 source,^27^ following clinical practice. Optimization was performed on 4.000 dose‐calculation points per organ. The resulting fronts were re‐evaluated on 20.000 dose‐calculation points per organ. These are the numbers of dose‐calculation points with which the bi‐objective dwell time optimization approach was introduced and validated,^12^, ^25^ and for which the difference between optimized and re‐evaluated values was shown to be small.^5^ For healthy tissue, optimization was already performed on 20.000 dose‐calculation points, to avoid slight violations of the constraint during re‐evaluation. The healthy tissue was defined as the smallest parallelepiped containing all active dwell positions in the catheter grid, with an added 5 mm margin, excluding all delineated organs (and tumor).

All considered algorithms have a stochastic component. Moreover, for each organ, the set of dose‐calculation points used for optimization is randomly sampled. The resulting fronts thus have a small dependency on the initial random seed used. Therefore, to increase reproducibility, all results shown are the median of 11 runs. Previous work shows that a runtime of GOMEA dwell time optimization of 30 s is sufficient for optimization in clinical practice.^5^ Since the healthy tissue constraint doubles the number of dose‐calculation points, GOMEA dwell time optimization is run for 1 min. GOMEA simultaneous catheter position and dwell time optimization is run for 5 min. Both CVT and GOMEA are run for each patient for 8, 10, 12, 14, and 16 catheters, where 16 is the number of catheters most frequently used in clinical practice at the Amsterdam UMC. For comparison, GOMEA dwell time optimization is also performed on the catheters that were used in the clinical treatment plans.

The Golden Corner^25^ is defined as the area where LCI>0 and LSI>0. To allow for a simple, yet meaningful comparison between GOMEA simultaneous catheter position and dwell time optimization, and CVT catheter position optimization followed by GOMEA dwell time optimization, each front is described by a single value L, based on the treatment plan on the front that is closest to the Golden Corner in both LCI and LSI. Specifically, for each patient and number of catheters,

$$L\;:=\underset{\text{fronts j}=1,\ldots ,11}{\text{median}}\left\{\max_{\text{plans i in front j}}\left\{\min\left\{{\text{LCI}}_{i},{\text{LSI}}_{i}\right\}\right\}\right\}$$

is used. If L>0, then the median front contains treatment plans satisfying all planning‐aims. Hence, if L>0, then the median front goes through the Golden Corner. If ‐1<L≤0, then we say that the median front came within 1% of the Golden Corner.

### 3.E Statistical tests

The difference between GOMEA simultaneous catheter position and dwell time optimization, and CVT catheter position optimization followed by GOMEA dwell time optimization, was tested with a Wilcoxon signed‐rank test on L for 16, 14, 12, 10, and 8 catheters separately.

## 4 RESULTS

**Fig. 2:**
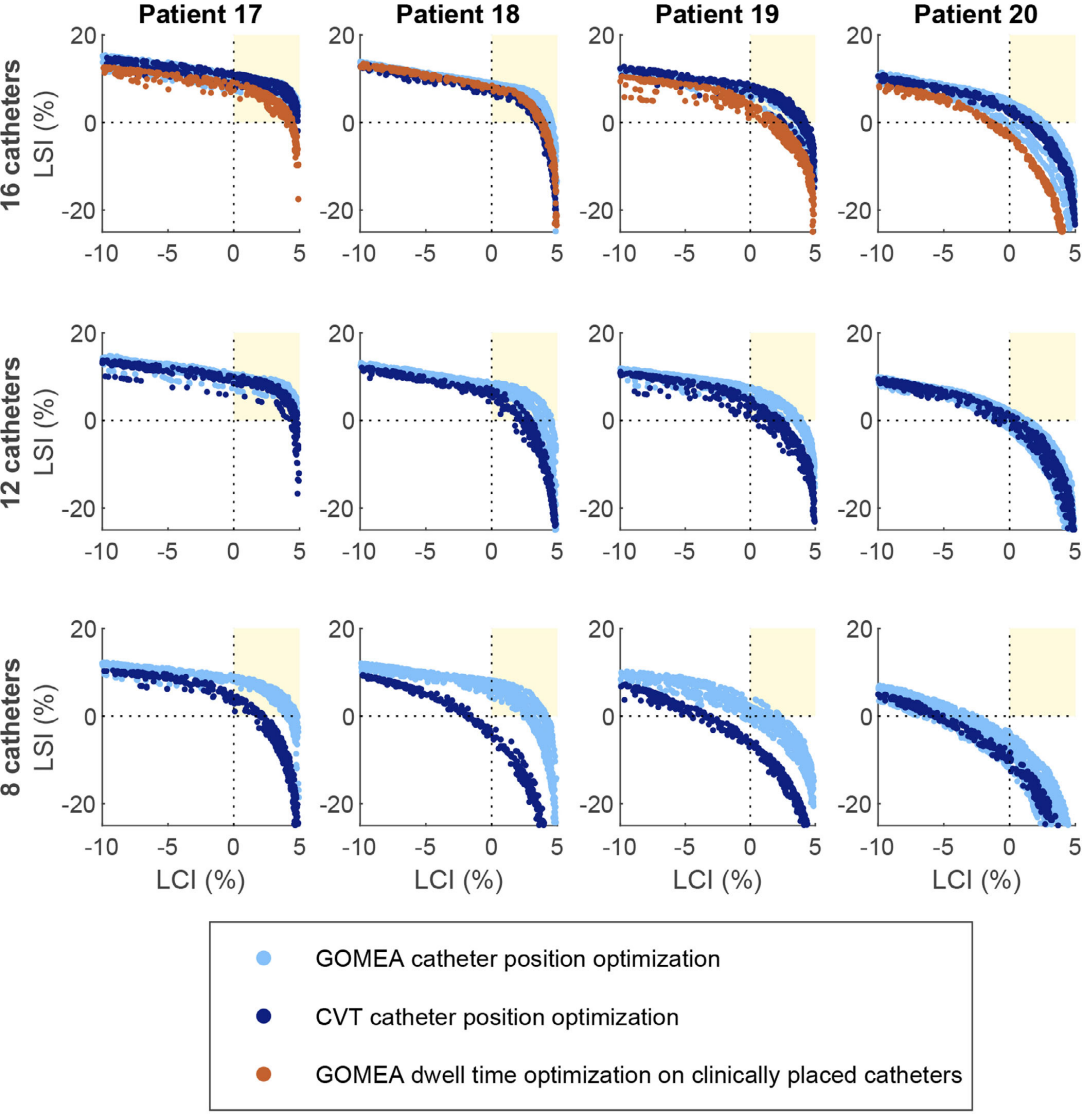
Results for four patients (columns) for three numbers of catheters (rows) using either GOMEA simultaneous catheter position and dwell time optimization (light blue), Centroidal Voronoi Tessellations catheter position optimization followed by GOMEA dwell time optimization (dark blue), or the 16 clinical catheters in combination with GOMEA dwell time optimization (brown). (Color versions of graphs are available online.) The Golden Corner is defined as the area where both objectives Least Coverage Index (LCI) and Least Sparing Index (LSI) are greater than zero. Each front can be described by a single value L, based on the treatment plan on the front that is closest to the Golden Corner in both LCI and LSI. The front either reached the Golden Corner (0<L), came within 1% of the Golden Corner (‐1<L≤0), or was at least 1% away from the Golden Corner (L≤‐1). [Color figure can be viewed at wileyonlinelibrary.com]

**Fig. 3:**
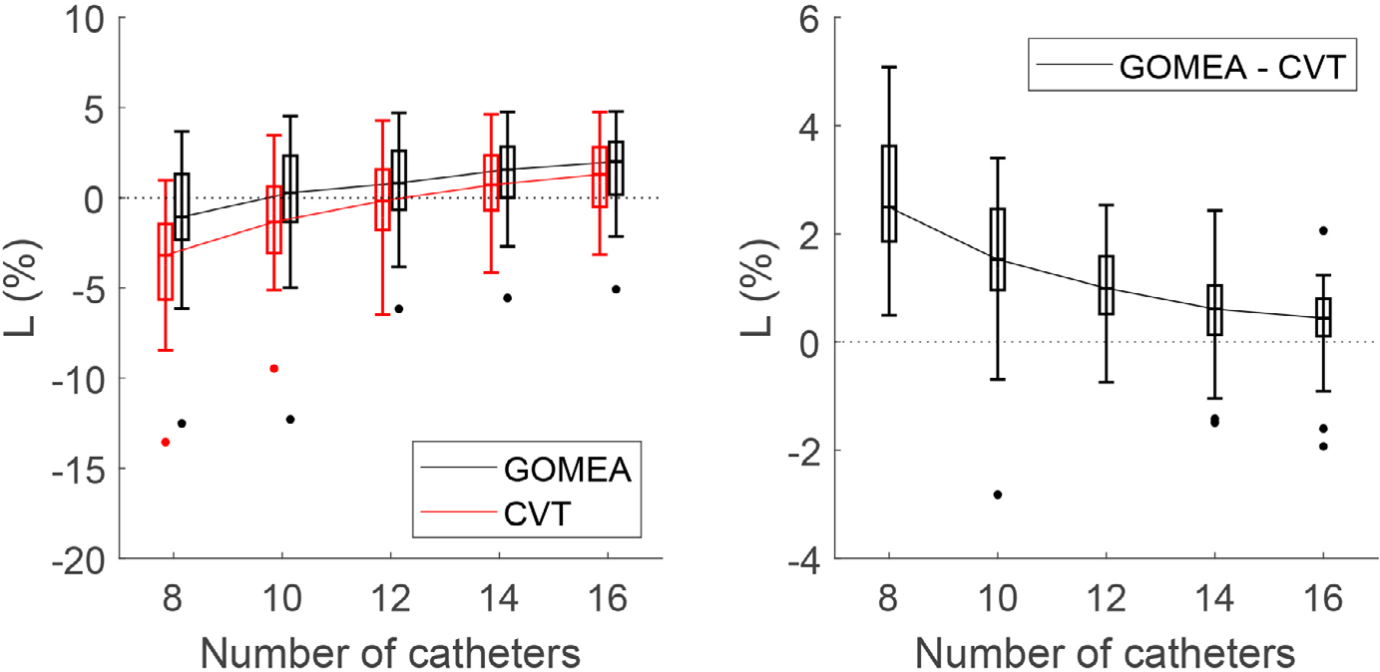
The results for GOMEA simultaneous catheter position and dwell time optimization, and Centroidal Voronoi Tessellations (CVT) catheter position optimization followed by GOMEA dwell time optimization, for 8, 10, 12, 14, and 16 catheters. For each patient, the median over 11 runs is taken, after which a boxplot over all patients is shown (median at 50%, box from 25% to 75%, whiskers at 0% and 100%, excluding outliers based on 1.5 times the interquartile range). The left figure shows the values of L for both GOMEA and CVT, each moved slightly to either left or right for improved visualization. The right figure shows the difference between GOMEA and CVT. [Color figure can be viewed at wileyonlinelibrary.com]

**Fig. 4:**
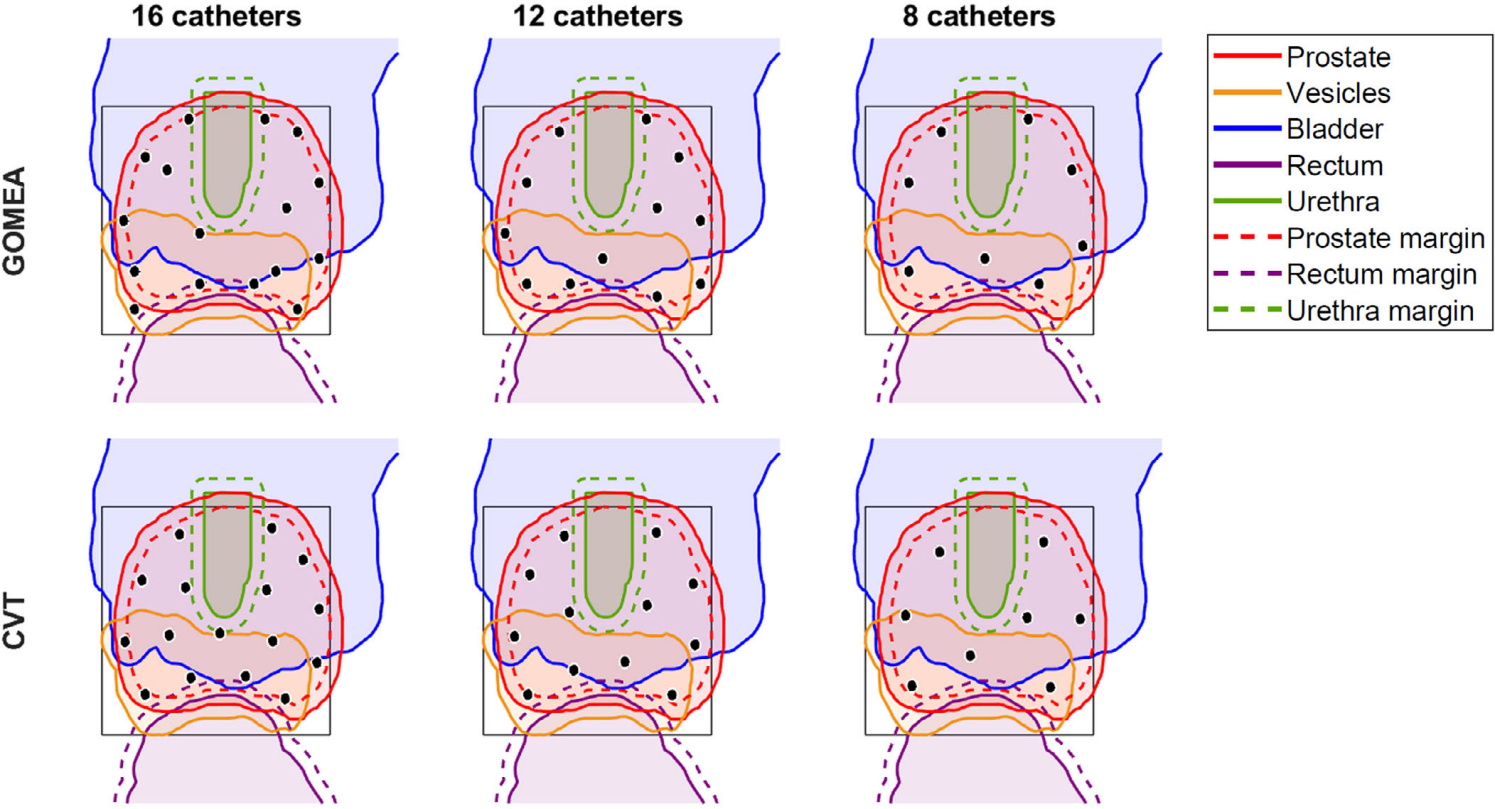
Organ projections with the catheter positions for GOMEA and Centroidal Voronoi Tessellations for 16, 12, and 8 catheters (for patient 18) corresponding to the value L. The black rectangles correspond to the catheter grids used by GOMEA, the black dots are the catheter positions. [Color figure can be viewed at wileyonlinelibrary.com]

A few typical examples of the resulting fronts are visualized in Fig. 2. The results for L are shown in Fig. 3 (full tables are in the appendix, as well as figures for the different dose‐volume indices). A few typical examples of the resulting catheter positions are visualized in Fig. 4.

For 16 catheters, GOMEA was able to find treatment plans satisfying all planning‐aims for 20 out of the 26 patients, and the front came within 1% of the Golden Corner for two other patients. CVT reached the Golden Corner for 19 patients and came within 1% for three other patients. The *P*‐value for the difference between the values of L for the two catheter position optimization methods was 0.023.

**Fig. 5:**
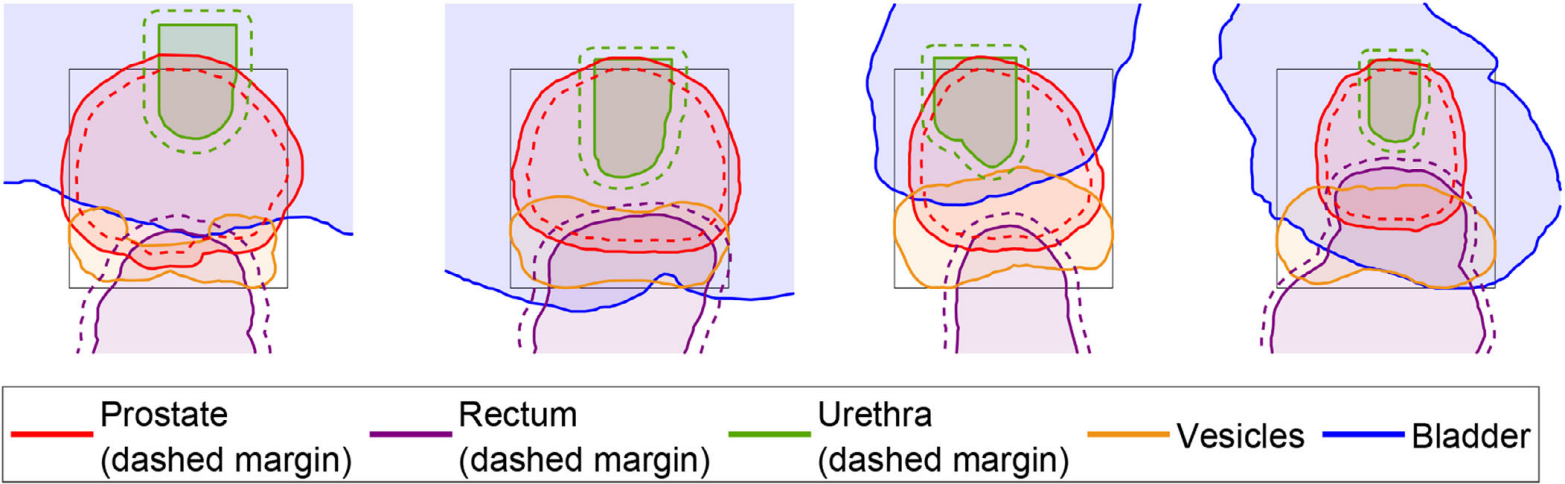
For four patients (7, 13, 24, and 25), with 16 catheters, GOMEA was not able to find treatment plans satisfying all planning‐aims within 1%. As can be seen in the organ projections, the rectum shape at the time of scanning often prevents catheters from reaching a large part of the seminal vesicles, which may not have been the case at the time of actual clinical catheter placement. The black rectangles correspond to the catheter grids used by GOMEA. [Color figure can be viewed at wileyonlinelibrary.com]

There are four patients for whom GOMEA was not able to come within 1% of the Golden Corner with 16 catheters, their organ projections are shown in Fig. 5. Although the convergence is slowest for such patients, 5 min of optimization was still sufficient (see also Figure A2 in the appendix). For these patients, the organ shape and position at the time of MRI scanning may not have been identical to those at the time of catheter placement, due to the difference in posture and the absence of the rectum probe. In particular, the clinical catheters were placed in a way that at the time of MRI scanning would have intersected the rectum. For patient 25, the large standard deviation in the values of L for GOMEA catheter position optimization (up to 4.79%) indicates a premature convergence of the optimization.

For 14 catheters, GOMEA was able to find treatment plans satisfying all planning‐aims for 20 out of the 26 patients, and the front came within 1% of the Golden Corner for one patient. CVT reached the Golden Corner for 17 patients and came within 1% for three patients. The *P*‐value for the difference between the values of L for the two catheter position optimization methods was 0.005.

For 12, 10, and 8 catheters, GOMEA was able to find treatment plans satisfying all planning‐aims for 18, 14, and 11 of the 26 patients, and the front came within 1% of the Golden Corner for two, four, and two patients. CVT reached the Golden Corner for 13, nine, and two patients and came within 1% for four, three, and two patients. For each of these numbers of catheters, the *P*‐value for the difference between the values of L for the two catheter position optimization methods was <0.001.

## 5 DISCUSSION

The results show that both GOMEA and CVT can obtain catheter positions in a clinically feasible runtime, where GOMEA takes 5 min. CVT has not been optimized for runtime, but it has already been reported to be able to obtain 10 catheter configurations ranging from 9 to 18 catheters in <10 s.^17^ One advantage of GOMEA is that the resulting front contains multiple configurations of catheter positions, where the final decision is left to a radiation oncologist. In contrast, CVT only provides a single configuration of catheter positions. Both methods require the number of catheters as input, which could be based on experience for previous patients. Additional experience could retrospectively be gained by running the optimization for multiple numbers of catheters.

The bi‐objective optimization model for dwell time optimization has been validated in an observer study with radiation oncologists.^12^ For bi‐objective catheter position optimization, treatment plans have previously been discussed with a radiation oncologist as well^21^ (see also appendix), resulting in the additional constraints to the healthy tissue and additional margins to the organ projections, both of which were developed in an iterative process together with the radiation oncologist. Therefore, we are confident about the clinical feasibility of the treatment plans.

Simultaneous optimization as done by GOMEA is slower than separate optimization as done by CVT catheter position optimization in combination with GOMEA dwell time optimization, due to the larger optimization problem. The longer runtime is however associated with better results: catheter positions obtained with GOMEA were better than those obtained with CVT. This advantage increased when the number of catheters decreased. For the use of 16 catheters that is currently clinical practice in our clinic, the added value in performing simultaneous optimization is very small. However, simultaneous optimization shows the potential to reduce the number of catheters used in clinical practice. Depending on the individual patient anatomy, possibly a reduction to as low as 8 catheters appeared feasible. The use of <14 catheters has been significantly associated with reduced toxicity.^28^ However, plans with lower numbers of catheters are also expected to have a lower robustness.

Robustness was not considered in the experiments. For CVT, it has been shown that the catheter positions are robust to small perturbations in random directions in the insertion plane if afterwards the dwell times are re‐optimized,^17^ at least in combination with IPSA dwell time optimization. It is therefore a reasonable assumption that this is also valid for CVT in combination with GOMEA dwell time optimization. However, perturbations in the depth to which each catheter was placed were not tested. For GOMEA, no robustness analysis to catheter perturbations has been performed yet.^21^ It would be of high practical value to be able to use robust optimization, to search for catheter positions that are robust to small perturbations.

Another limitation of this study is that the patient data used in the experiments was obtained only after the catheter placement, and in a different posture. At the time of catheter placement, the patient was in the operating room in the lithotomy position and an ultrasound was used. For acquisition of MRI scans for a final check of the catheter positions and the treatment planning, the patient was moved to an MRI scanner and positioned in supine position during scanning. Therefore, the organs at the time of scanning may not have been representative of the organs at the time of catheter placement, which is when in clinical practice the optimization would take place. A few patients were already highlighted for this reason, for whom none of the optimized treatment plans satisfied all planning‐aims. For future work, the use of actual patient data from the time of catheter placement would be preferable.

Finally, the optimization model was based on the clinical protocol used at the Amsterdam UMC. Combining dose indices and volume indices into a single objective worked well for this protocol, but this may not be the case in general. For future work, involving other protocols, normalization of the dose‐volume indices may be required.

## 6 CONCLUSIONS

With bi‐objective simultaneous optimization of catheter positions and dwell times, high‐quality catheter positions optimized directly for dose‐volume indices of clinical interest can now be obtained within a clinically feasible runtime of 5 min. Especially for lower numbers of catheters, better plan quality can be achieved compared to the use of the state‐of‐the‐art CVT approach.

## CONFLICTS OF INTEREST

This work was partly funded by Elekta AB (Stockholm, Sweden). Dr. Alderliesten, Dr. Bel, Prof. Dr. Bosman, and Dr. Pieters are involved in projects supported by Elekta. Yury Niatsetski is an Elekta employee.

## Supporting information


**Data S1**. The Supporting Information consists of our investigations for the healthy tissue constraint, our investigations into the convergence of the optimization, figures of the different dose‐volume indices, and full tables of the results.
