# Supplementary material for: Bi‐objective optimization of catheter positions for high‐dose‐rate prostate brachytherapy
Source: Med Phys. 2020 Oct 21;47(12):6077–86. doi: 10.1002/mp.14505 (PMC7821293; doi:10.1002/mp.14505)
Supplement: Supplementary file 1 — Data S1. The Supporting Information consists of our investigations for the healthy tissue constraint, our investigations into the convergence of the optimization, figures of the different dose‐volume indices, and full tables of the results. [file MP-47-6077-s001.docx]

**Bi-objective optimization of catheter positions for high-dose-rate prostate brachytherapy**

Supplementary Material

**Additional experiments**

We extended the catheter position optimization model by defining an additional constraint $C=0.125*N-V_{200\%}^{healthy tissue}$, where $N$ is the number of catheters, with $C\geq0.$ The lower the number of catheters, the lower the value of $C$, hence the stricter the constraint to the dose to healthy tissue. Both the original and the extended model were tested on data of 6 prostate cancer patients treated with high-dose-rate brachytherapy. To test the effectiveness, for 24 pairs of plans for 6 patients and 4 different numbers of catheters from each model, the 3D isodose distributions were visually evaluated together with a radiation oncologist.

Figure A1 shows the fronts of optimized plans, together with the 24 selected pairs of plans. All 24 plans from the new model were visually evaluated as feasible in terms of healthy tissue dose. Moreover, 22 plans from the new model were deemed superior to the corresponding 22 plans from the original optimization model.


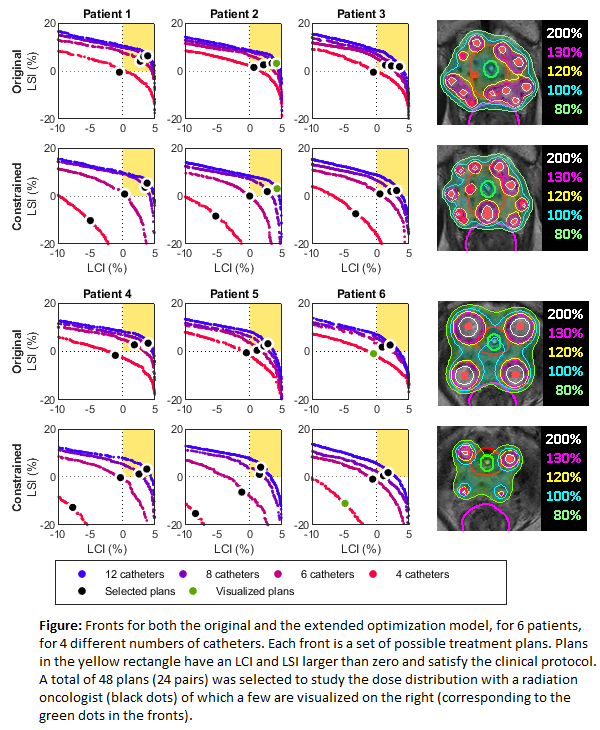


Figure A1. Fronts for both the original and the extended optimization model, for 6 patients, for 4 different numbers of catheters. Each front is a set of possible treatment plans. Plans in the yellow rectangle have an LCI and LSI larger than zero and satisfy the clinical protocol. A total of 48 plans (24 pairs) was selected to study the dose distribution with a radiation oncologist (black dots) of which a few are visualized on the right (corresponding to the green dots in the fronts).


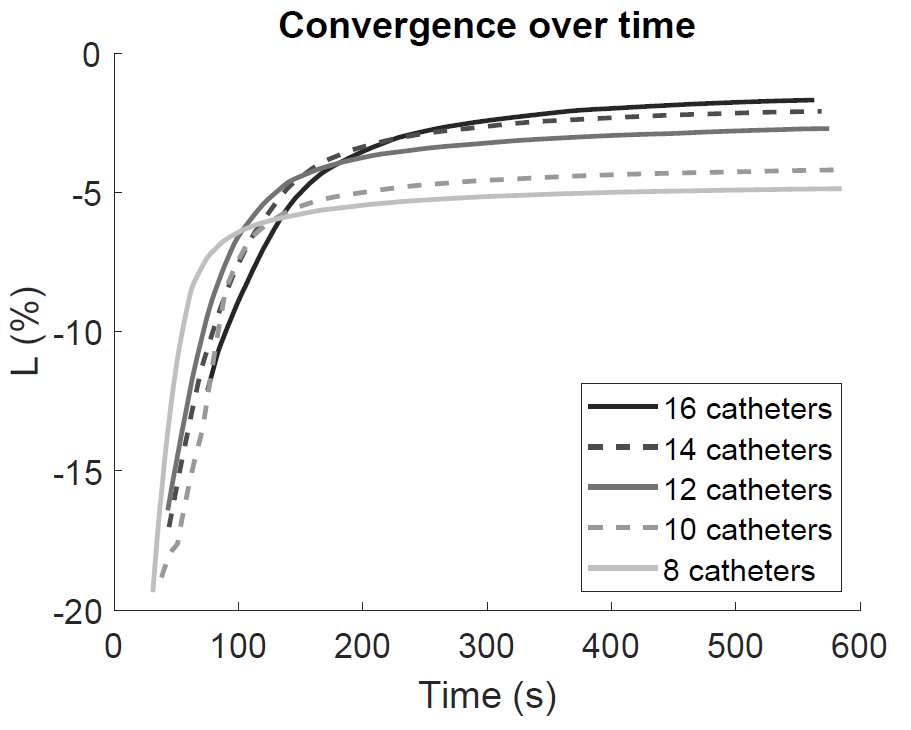


Figure A2: The convergence of the results for GOMEA simultaneous catheter position and dwell time optimization for 8, 10, 12, 14, and 16 catheters. The Golden Corner is defined as the area where both objectives LCI and LSI are greater than zero. Each front is described by a single value L, based on the treatment plan on the front that is closest to the Golden Corner in both LCI and LSI. The convergence of L is averaged over 11 runs of patients 7, 13, 24, and 25.


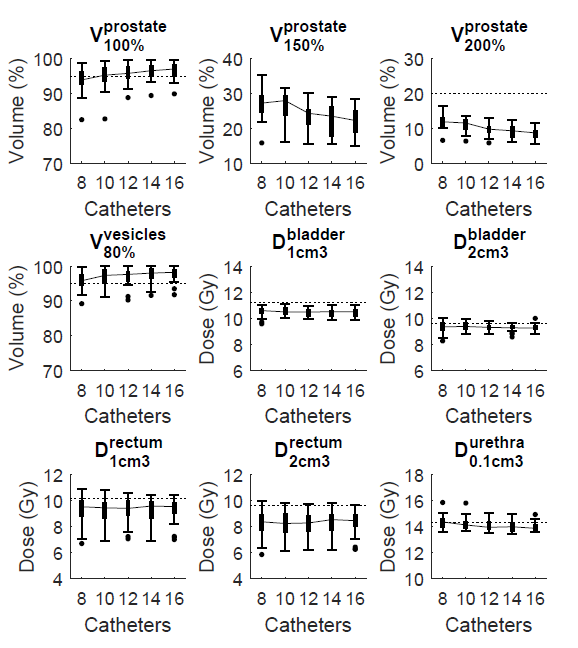


Figure A3. The results for GOMEA simultaneous catheter position and dwell time optimization, for 8, 10, 12, 14, and 16 catheters. The Golden Corner is defined as the area where both objectives LCI and LSI are greater than zero. Each front is described by a single value L, based on the treatment plan on the front that is closest to the Golden Corner in both LCI and LSI. For each patient, the median of L over 11 runs is taken, after which a boxplot over all patients is shown for all dose-volume indices separately.

|  | GOMEA, L (standard deviation) (%) | | | | |
| --- | --- | --- | --- | --- | --- |
| Patient | 8 | 10 | 12 | 14 | 16 |
| 1 | **1.32(0.33)** | **2.11(0.45)** | **2.33(0.36)** | **2.43(0.25)** | **2.41(0.18)** |
| 2 | **2.72(0.50)** | **3.95(0.29)** | **4.15(0.14)** | **4.18(0.22)** | **4.21(0.14)** |
| 3 | **0.99(0.44)** | **1.59(0.42)** | **1.99(0.36)** | **2.39(0.39)** | **2.33(0.28)** |
| 4 | -2.52(0.52) | -1.07(0.55) | -0.05(0.64) | **0.61(0.73)** | **0.92(0.52)** |
| 5 | -1.61(0.42) | -0.41(0.72) | **0.63(0.52)** | **1.20(0.78)** | **2.07(0.66)** |
| 6 | -1.16(0.66) | -0.13(0.34) | **0.37(0.16)** | **0.52(0.41)** | **0.74(0.26)** |
| 7 | -1.67(0.29) | -1.34(0.17) | -1.17(0.27) | -1.14(0.10) | -1.16(0.23) |
| 8 | -0.74(0.63) | **0.49(0.57)** | **0.31(0.64)** | **0.69(0.38)** | **0.99(0.45)** |
| 9 | -1.41(0.87) | **0.06(1.86)** | **0.60(2.13)** | **1.64(0.79)** | **1.94(0.48)** |
| 10 | **3.69(0.23)** | **4.52(0.21)** | **4.69(0.22)** | **4.76(0.09)** | **4.79(0.06)** |
| 11 | -3.58(0.49) | -2.49(0.31) | -1.64(0.37) | -1.21(0.71) | -0.01(0.68) |
| 12 | **0.89(0.40)** | **1.92(0.22)** | **2.54(0.39)** | **2.66(0.20)** | **2.62(0.26)** |
| 13 | -6.14(0.68) | -4.98(0.48) | -3.84(0.27) | -2.70(0.44) | -2.13(0.30) |
| 14 | **0.34(0.49)** | **1.85(0.40)** | **2.61(0.48)** | **2.83(0.26)** | **3.11(0.18)** |
| 15 | **2.18(0.34)** | **2.78(0.27)** | **3.00(0.24)** | **2.98(0.24)** | **3.06(0.20)** |
| 16 | **2.39(0.39)** | **3.14(0.26)** | **3.35(0.23)** | **3.49(0.14)** | **3.58(0.25)** |
| 17 | **3.50(0.34)** | **4.41(0.20)** | **4.65(0.16)** | **4.58(0.16)** | **4.72(0.19)** |
| 18 | **2.39(0.58)** | **2.99(0.61)** | **3.11(0.53)** | **3.81(0.29)** | **3.78(0.23)** |
| 19 | **0.49(0.53)** | **2.32(0.53)** | **2.45(0.63)** | **2.76(0.33)** | **2.82(0.49)** |
| 20 | -2.26(0.81) | -0.91(0.64) | **0.03(0.60)** | **0.91(0.71)** | **1.25(0.76)** |
| 21 | -0.97(0.55) | **1.21(0.44)** | **2.05(0.39)** | **2.78(0.53)** | **3.22(0.27)** |
| 22 | -1.79(0.61) | -0.32(0.49) | **1.00(0.44)** | **1.49(0.50)** | **1.83(0.45)** |
| 23 | -4.28(0.88) | -2.14(1.35) | -0.66(0.75) | **0.01(0.84)** | **0.16(0.46)** |
| 24 | -2.32(0.72) | -1.72(0.17) | -1.52(0.12) | -1.48(0.11) | -1.46(0.18) |
| 25 | -12.51(3.92) | -12.29(4.08) | -6.16(4.79) | -5.56(4.03) | -5.08(3.48) |
| 26 | -4.33(0.67) | -2.34(0.87) | -1.20(0.73) | -0.52(0.42) | -0.30(0.30) |

Table A1. The results for GOMEA simultaneous catheter position and dwell time optimization for 8, 10, 12, 14, and 16 catheters. The Golden Corner is defined as the area where both objectives LCI and LSI are greater than zero. Each front is described by a single value L, based on the treatment plan on the front that is closest to the Golden Corner in both LCI and LSI. The front either reached the Golden Corner (bold), came within 1% of the Golden Corner (underlined), or was at least 1% away from the Golden Corner (normal). The table shows in brackets the standard deviation over 11 runs.

|  | CVT, L (standard deviation) (%) | | | | | Clinical plan | |
| --- | --- | --- | --- | --- | --- | --- | --- |
| Patient | 8 | 10 | 12 | 14 | 16 | N | L |
| 1 | -2.48(0.78) | **0.05(0.80)** | **0.46(0.38)** | **1.38(0.56)** | **2.16(0.36)** | 14 | **1.16(0.34)** |
| 2 | -0.90(0.54) | **1.23(0.49)** | **2.64(0.31)** | **3.19(0.34)** | **3.68(0.29)** | 14 | **3.99(0.26)** |
| 3 | -3.15(0.69) | -0.44(0.53) | **0.41(0.65)** | **0.92(0.62)** | **1.65(0.35)** | 16 | -0.45(0.38) |
| 4 | -6.52(0.86) | -3.92(0.40) | -2.58(0.40) | -1.82(0.52) | -1.14(0.24) | 15 | **2.28(0.20)** |
| 5 | -4.47(0.82) | -1.61(0.71) | **0.03(0.76)** | **0.59(0.30)** | **1.24(0.28)** | 16 | -6.36(0.48) |
| 6 | -3.93(0.31) | -2.29(0.44) | -1.14(0.49) | -0.69(0.43) | -0.50(0.50) | 14 | -1.28(0.51) |
| 7 | -4.13(0.48) | -2.74(0.45) | -1.92(0.28) | -1.66(0.34) | -1.46(0.44) | 16 | -4.89(0.53) |
| 8 | -3.08(0.58) | -1.89(0.71) | -0.64(0.34) | -0.29(0.56) | **0.22(0.41)** | 17 | **1.86(0.39)** |
| 9 | -2.54(0.30) | -1.07(0.46) | -0.46(0.38) | **0.82(0.25)** | **1.35(0.49)** | 17 | **1.42(0.25)** |
| 10 | **0.16(0.92)** | **2.86(0.22)** | **4.28(0.29)** | **4.63(0.11)** | **4.75(0.09)** | 14 | **2.26(0.41)** |
| 11 | -5.65(0.72) | -3.23(0.61) | -1.79(0.67) | -0.17(0.42) | **0.68(0.47)** | 15 | **0.40(0.31)** |
| 12 | -3.21(0.63) | -0.56(0.60) | **0.56(0.86)** | **1.54(0.51)** | **1.79(0.48)** | 17 | **0.06(0.35)** |
| 13 | -8.45(1.44) | -4.29(0.71) | -3.10(0.56) | -1.21(0.39) | -0.53(0.37) | 14 | **0.80(0.30)** |
| 14 | -1.27(0.62) | **0.67(0.28)** | **1.57(0.33)** | **2.34(0.25)** | **2.64(0.19)** | 16 | **1.89(0.25)** |
| 15 | -0.17(0.48) | **1.57(0.37)** | **2.05(0.37)** | **2.54(0.25)** | **2.79(0.23)** | 17 | -0.78(0.33) |
| 16 | -1.14(0.60) | **0.63(0.40)** | **1.47(0.65)** | **2.96(0.35)** | **3.17(0.35)** | 18 | **3.18(0.50)** |
| 17 | **0.98(0.47)** | **3.45(0.46)** | **4.13(0.29)** | **4.47(0.25)** | **4.61(0.19)** | 16 | **3.42(0.32)** |
| 18 | -1.69(0.27) | **0.53(0.36)** | **1.58(0.46)** | **2.21(0.24)** | **2.81(0.32)** | 16 | **3.11(0.15)** |
| 19 | -2.47(0.62) | -0.27(0.50) | **1.06(0.48)** | **2.15(0.84)** | **2.89(0.46)** | 16 | **1.07(0.56)** |
| 20 | -3.81(0.47) | -1.67(0.81) | -0.37(0.53) | **0.45(0.19)** | **0.45(0.37)** | 16 | -1.23(0.27) |
| 21 | -1.46(0.35) | **0.74(0.42)** | **1.86(0.51)** | **2.78(0.16)** | **3.11(0.21)** | 18 | -0.20(0.33) |
| 22 | -3.65(1.08) | -1.97(0.55) | -0.63(0.50) | **0.12(0.35)** | **0.89(0.34)** | 17 | -0.89(0.35) |
| 23 | -6.35(1.27) | -3.08(0.85) | -1.21(0.53) | **0.08(0.65)** | **1.07(0.56)** | 19 | **2.07(0.39)** |
| 24 | -7.40(0.31) | -5.12(0.40) | -3.81(0.53) | -2.39(0.41) | -1.66(0.25) | 18 | **4.52(0.13)** |
| 25 | -13.55(1.02) | -9.47(1.45) | -6.48(1.23) | -4.14(1.47) | -3.15(0.59) | 17 | **3.42(0.24)** |
| 26 | -6.12(0.38) | -3.72(0.62) | -2.14(0.22) | -1.49(0.20) | -0.85(0.31) | 20 | -0.84(0.24) |

Table A2. The results for CVT catheter position optimization for 8, 10, 12, 14, and 16 catheters followed by GOMEA dwell time optimization, and GOMEA dwell time optimization on the clinical catheters (column with N), ranging from 14 to 20 catheters. The Golden Corner is defined as the area where both objectives LCI and LSI are greater than zero. Each front is described by a single value L, based on the treatment plan on the front that is closest to the Golden Corner in both LCI and LSI. The front either reached the Golden Corner (bold), came within 1% of the Golden Corner (underlined), or was at least 1% away from the Golden Corner. The table shows in brackets the standard deviation over 11 runs.
